# Procalcitonin guided antibiotic therapy and hospitalization in patients with lower respiratory tract infections: a prospective, multicenter, randomized controlled trial

**DOI:** 10.1186/1472-6963-7-102

**Published:** 2007-07-05

**Authors:** Philipp Schuetz, Mirjam Christ-Crain, Marcel Wolbers, Ursula Schild, Robert Thomann, Claudine Falconnier, Isabelle Widmer, Stefanie Neidert, Claudine A Blum, Ronald Schönenberger, Christoph Henzen, Thomas Bregenzer, Claus Hoess, Martin Krause, Heiner C Bucher, Werner Zimmerli, Beat Müller, the ProHOSP study group

**Affiliations:** 1 Department of Internal Medicine and Department of Endocrinology, Diabetes and Clinical Nutrition, University Hospital Basel, Petersgraben 4, 4031 Basel, Switzerland; 2 Basel Institute of Clinical Epidemiology (BICE), University Hospital Basel, Petersgraben 4, 4031 Basel, Switzerland; 3 Departement of Internal Medicine Bürgerspital Solothurn, Schöngrünstrasse 42, 4500 Solothurn, Switzerland; 4 Departement of Internal Medicine Kantonsspital Liestal, Rheinstrasse 26, 4410 Liestal, Switzerland; 5 Departement of Internal Medicine Kantonsspital Aarau, Tellstrasse, 5001 Aarau, Switzerland; 6 Departement of Internal Medicine Kantonsspital Luzern, Spitalstrasse, 6000 Luzern 16, Switzerland; 7 Departement of Internal Medicine Kantonsspital Münsterlingen, 8596 Münsterlingen, Switzerland

## Abstract

**Background:**

Lower respiratory tract infections like acute bronchitis, exacerbated chronic obstructive pulmonary disease and community-acquired pneumonia are often unnecessarily treated with antibiotics, mainly because of physicians' difficulties to distinguish viral from bacterial cause and to estimate disease-severity. The goal of this trial is to compare medical outcomes, use of antibiotics and hospital resources in a strategy based on enforced evidence-based guidelines versus procalcitonin guided antibiotic therapy in patients with lower respiratory tract infections.

**Methods and design:**

We describe a prospective randomized controlled non-inferiority trial with an open intervention. We aim to randomize over a fixed recruitment period of 18 months a minimal number of 1002 patients from 6 hospitals in Switzerland. Patients must be >18 years of age with a lower respiratory tract infections <28 days of duration. Patients with no informed consent, not fluent in German, a previous hospital stay within 14 days, severe immunosuppression or chronic infection, intravenous drug use or a terminal condition are excluded. Randomization to either guidelines-enforced management or procalcitonin-guided antibiotic therapy is stratified by centre and type of lower respiratory tract infections. During hospitalization, all patients are reassessed at days 3, 5, 7 and at the day of discharge. After 30 and 180 days, structured phone interviews by blinded medical students are conducted. Depending on the randomization allocation, initiation and discontinuation of antibiotics is encouraged or discouraged based on evidence-based guidelines or procalcitonin cut off ranges, respectively. The primary endpoint is the risk of combined disease-specific failure after 30 days. Secondary outcomes are antibiotic exposure, side effects from antibiotics, rate and duration of hospitalization, time to clinical stability, disease activity scores and cost effectiveness. The study hypothesis is that procalcitonin-guidance is non-inferior (i.e., at worst a 7.5% higher combined failure rate) to the management with enforced guidelines, but is associated with a reduced total antibiotic use and length of hospital stay.

**Discussion:**

Use of and prolonged exposure to antibiotics in lower respiratory tract infections is high. The proposed trial investigates whether procalcitonin-guidance may safely reduce antibiotic consumption along with reductions in hospitalization costs and antibiotic resistance. It will additionally generate insights for improved prognostic assessment of patients with lower respiratory tract infections.

**Trial registration:**

ISRCTN95122877

## Background

Lower respiratory tract infections (LRTI) including acute bronchitis, acute exacerbations of chronic obstructive pulmonary disease (AECOPD) and community-acquired pneumonia (CAP), account for almost 10% of the worldwide burden of morbidity and mortality, and thus, importantly contribute to antibiotic overuse and allocation of health resources [1,2]. Although most LRTI are of viral origin, approximately 75% of antibiotics are prescribed because viral etiology and disease severity are difficult to recognize with traditional clinical and laboratory means [2]. Current criteria for assessing the severity of CAP, such as the pneumonia severity index (PSI) and the CAP severity on presentation to hospital scale (CURB65) are well validated, but have important drawbacks for routine care [3-6]. They only predict mortality in CAP and depend mainly on age, thereby underestimating the disease-related morbidity in younger patients. Furthermore, they dichotomize continuously measured values (e.g. respiratory rate) into normal and abnormal values and show a high intra-observer variation of around 10 percent.

A widely used approach to estimate the probability of a bacterial origin and the disease severity of a LRTI is the use of the C-reactive protein. However, this biomarker lacks sensitivity and specificity, and thorough studies about its effect on antibiotic use are lacking [7,8]. More promising appears to be the level of circulating procalcitonin (PCT) which has been demonstrated to correlate with the likelihood for a bacterial infection [7,9]. We conceived and successfully validated PCT guided diagnosis using cut-off ranges in the continuum of LRTI in four intervention trials by randomizing patients to PCT guided antibiotic prescription versus standard care and monitored clinical outcome. In these trials we circumvented the gold standard dilemma for the etiologic diagnosis of LRTIs by assuming absence of serious bacterial infection in patients recovering without antibiotics [10-14].

Some limitations of the previous intervention trials need to be considered. Three of the four trials were conducted at a single University Hospital, limiting the external validity of this approach [11,12,14]. Routine use of guidelines for antibiotic prescriptions was only partly enforced in the standard care group [11-14]. Only the primary care trial had adequate power to show non-inferiority in days with restrictions from LRTI by PCT guidance. However, the trial was not designed to show non-inferiority from severe infectious disease complications, more typically seen in hospitalized patients with higher complication rates. Finally, despite a marked reduction of the duration of antibiotic therapy by PCT guidance, the length of hospital stay was not reduced since it was not a target of intervention in all previous trials.

The aim of the proposed "ProHOSP"-study is to address these limitations from the previous trials and to study additional diagnostic and prognostic biomarkers for the management of LRTIs. In addition this trial shall assess the impact of a biomarker driven LRTI management on hospital stay and costs.

## Methods

The objectives of this randomized controlled, open intervention trial are to evaluate whether a PCT guided diagnostic and therapeutic strategy in patients with LRTI lead to similar patient relevant outcomes, reduced total antibiotic use as well as length of hospitalization as compared to a management without PCT testing but based on the enforced implementation of current guidelines. The primary study hypothesis is that a PCT guided LRTI management is non-inferior to standard care based on implemented guidelines. The primary endpoint is disease specific failure within 30 days following index hospital admission and we assume non-inferiority if the combined disease specific failure rate is less than 7.5%.

Patients from six hospitals in Switzerland are being included. Full ethical approval for this trial which is in compliance with the Helsinki Declaration has been obtained from all local ethical committees. All responsible heads of the medical departments of the participating study hospitals have approved and signed the study protocol and all participating patients must give written informed consent.

This trial is supervised by an independent safety monitoring board which is not involved in the design and the conduct of the trial. The board consists of a pneumologist, an infectious disease specialist and an intensive care specialist.

### Setting

We recruit the patients in six secondary and tertiary care clinics in northern and central Switzerland. The characterisation of the study clinics is presented in table 1. The 6 public hospitals are variable in size, patient care capacity and employment of medical and nursing staff, but have a comparable length of hospital stay per patient (mean hospital stay 8.3 +/- 0.57 days)[15].

**Table 1:** Baseline characteristics of the six participating study hospitals in the northern and central part of Switzerland [15].

**Baseline characteristics of the study hospitals**						
	**Universitäts-spital Basel**	**Kantonsspital Liestal**	**Kantonsspital Luzern**	**Kantonsspital Aarau**	**Bürgerspital Solothurn**	**Kantonsspital Münsterlingen**

Status	Public	Public	Public	Public	Public	Public

Number of beds	694	371	616	539	254	252
Mean length of stay (days)	8.5	9.9	8.5	8.5	9.3	8.3
% of private medical coverage*	28.3	19.0	18.9	18.5	25.1	15
Medical staff**	849	125	414	376	97	350
Nursing staff**	1047	315	1074	979	417	414

*Average of medical and surgical patients

** All personal for the care of medical and surgical patients

Local investigators and their staff received a structured seminar to become familiar with the details of the protocol, the rationale and the design of the trial, the study website for patients inclusion and randomisation and all study forms.

In December 2006, all participating hospitals started to consecutively screen all adult patients admitted to the emergency department with suspected LRTI.

### Participants

Inclusion criteria for patients are written informed consent, age ≥ 18 years and admittance from the community or a nursing home with the main diagnosis of acute LRTI (i.e., less than 28 days). LRTI is defined by at least one respiratory symptom (cough, sputum production, dyspnea, tachypnea pleuritic pain) plus one auscultatory finding or sign of infection (core body temperature >38.0°C, shivers, leucocyte count >10 G/L or <4 G/L cells) independent of antibiotic pre-treatment. The LRTI conditions are defined as follows: CAP is defined as a new or increased infiltrate on chest radiograph [16,17]. COPD is defined by post-bronchodilator spirometric criteria according to the GOLD-guidelines as a FEV1/FVC ratio below 70% and the severity categorized according to GOLD criteria [18,19]. Acute bronchitis is defined as LRTI in the absence of an underlying lung disease or focal chest signs or infiltrates on chest X-ray, respectively [20]. Exclusion criteria are the inability to give written informed consent, insufficient German language skills, active intravenous drug users, severe immunosuppression, accompanying chronic infection or endocarditis or very severe medical co-morbidity where death is imminent.

### Intervention

**Figure 1:**
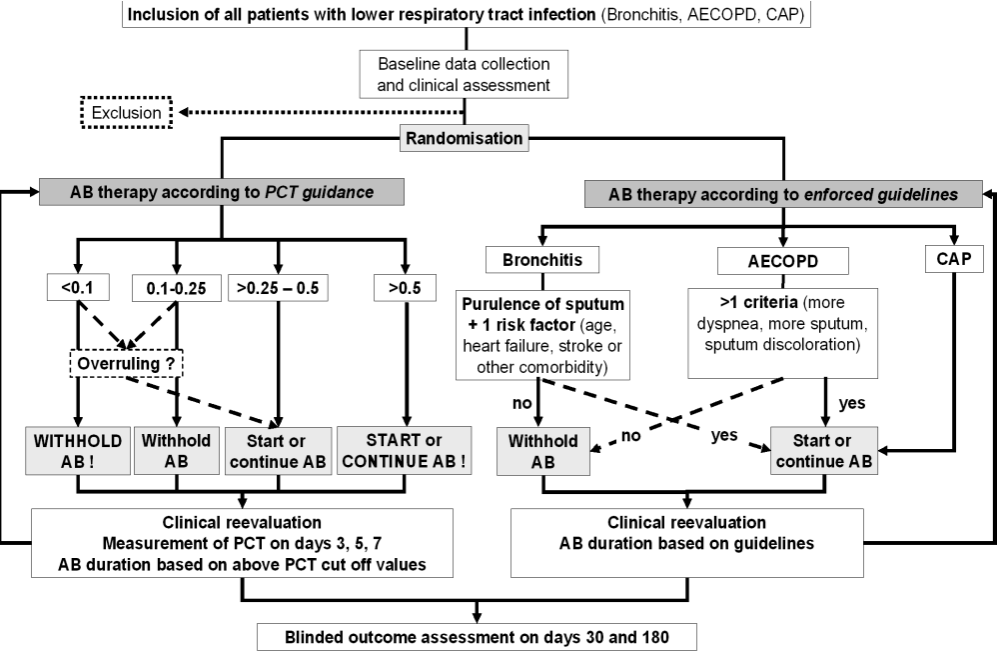
All consecutive patients with lower respiratory tract infection are potentially eligible for this trial. If all inclusion criteria are fulfilled and no exclusion criteria are present, the physician has to explain to the patient the trial, ask for participation and get informed consent. After inclusion, the patient is randomized by a web based computerized random allocation algorithm to either the guidelines group or the PCT group, respectively. *CAP *denotes community-acquired pneumonia, *AECOPD *acute exacerbation of chronic pulmonary disease, *AB *antibiotics, *PCT *procalcitonin.

Clinicians in the emergency departments of participating clinics are advised to access a web based study algorithm and enter baseline data of all eligible patients with LRTI on admission and to check all inclusion and exclusion criteria prior to randomisation (figure 1). Randomization of patients to PCT guidance or guideline enforced antibiotic therapy is based on a pre-specified computer generated randomization list and concealed by using a centralized password-secured website [21]. This website provides all study-related information including guidelines and patient flow. The randomization is stratified by the participating clinic and the type of LRTI (acute bronchitis, AECOPD, CAP).

We summarized guidelines on the management of CAP, acute bronchitis and AECOPD based on the most recent guidelines by the European Respiratory Society (ERS) and the American Thoracic Society (ATS) supplemented by evidence from recent guidelines and published current concepts [16,17,19,20]. These guidelines have been adapted by a panel of local internists, emergency physicians, pneumologists, infectious disease experts and clinical epidemiologists and have been successfully used in the clinical setting. To optimize the implementation of these guidelines for all patients the treating physician is enforced to follow web-based guideline algorithms, controlled by email alerts released for every patient screened and recruited, respectively. If the algorithm for PCT guidance or the guidelines for antibiotic therapy are overruled, the study centre has to be informed as soon as possible.

PCT is measured using a rapid sensitive immunoassay with a functional assay sensitivity of 0.06 ug/L (Kryptor PCT, Brahms, Hennigsdorf, Germany). The coefficient of variation of the assay at 0.1 ug/L, 0.25 ug/L, 0.5 ug/L and 10 ug/L were 16%, 7%, 5% and 3%, respectively. The test is performed at the central lab of each participating hospital. The assay time for PCT measurements require less than 20 minutes and PCT results are routinely available within one hour upon ordering.

**Figure 2:**
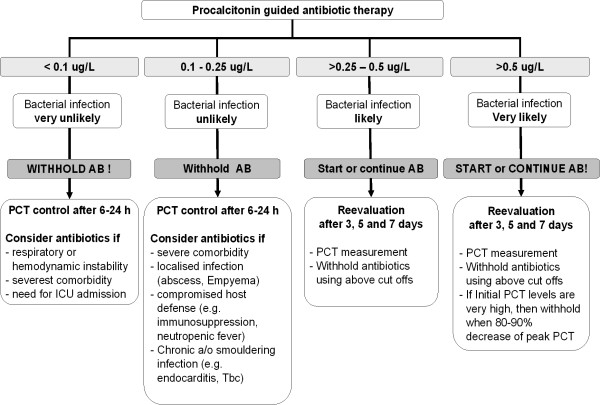
Antibiotic stewardship based on procalcitonin (PCT) cut-off ranges. Re-evaluation of the clinical status and measurement of serum PCT levels is mandatory after 6–24 h in all persistently sick and hospitalized patients in who antibiotic are withheld. The PCT algorithm can be overruled by pre-specified criteria, e.g. in patients with immediately life-threatening disease. If the algorithm is overruled and antibiotics are given, an early discontinuation of antibiotic therapy after 3, 5 or 7 days is more or less endorsed based on PCT levels. In hospitalized patients with ongoing antibiotic therapy PCT levels are reassessed on days 3, 5 and 7 and antibiotics will be discontinued using the PCT cut-offs defined above. In all patients with a very high PCT value on admission (e.g., >10 μg/L), discontinuation of antibiotic is already encouraged if levels decreased below 80 to 90% of the initial value. In patients discharged and, thus, likely uncomplicated resolution of the infection or in patients transferred to an institution not taking part in this trial the recommended total duration of antibiotic therapy is based on the last PCT level and is as following: >1 ug/L 7 days, 0.5–0.99 ug/L 5 days, 0.25–0.49 ug/L 3 days, <0.25 ug/L stop antibiotic, <0.1 ug/L STOP antibiotic. *PCT *denotes procalcitonin, *AB *antibiotics,*Tbc *tuberculosis, *ICU *intensive care unit,

PCT levels are communicated by the password secured website to the treating physician together with a treatment recommendation for antibiotics based on the PCT algorithm exclusively for patients randomized to the PCT intervention arm. Similarly, for the patients in the guideline-enforced group treatment recommendation based on guidelines are displayed. All participating physicians received detailed information about the use of PCT cut off ranges and the algorithm as presented in figure 2 is published in the investigators brochure and on the internet platform.

In both groups, hospitalized patients are clinically reassessed and blood is sampled on days 3, 5, 7, and on the day of discharge to assess the resolution of the presumed infection. In both groups, a switch of antibiotics from intravenous to oral is advised if patients show stable or improving vital signs, resolution of the predominant clinical sign or if oral intake is possible (adequate consciousness and reflexes to swallow fluids and tablets, no malabsorption) [16,17]. In hospitalized patients with an acute bronchitis, a chest X-ray after 3–5 days is routinely performed to confirm the diagnosis and exclude pneumonia.

In all hospitalized patients hospital discharge should be considered if oral intake is feasible, vital signs are stable > 24 h (as defined above), and no evidence of acute serious co-morbidity that necessitates hospitalization is present and, if the patient has achieved pre-admission mobility state [16,17]. At the day of discharge, all patients receive 2 leaflets providing general information for the patient and the general physician (GP) regarding this trial.

### Outcomes and adverse events

The primary endpoint of this trial is the combined disease-specific failure rate within 30 days. The following events are considered as failures: (**a**) radiologically, microbiologically or clinically confirmed recurrence of infection in need of antibiotics, (**b**) local or systemic complications from LRTI including persistence or development of pneumonia (including nosocomial), parapneumonic effusions, lung abscess, empyema, any abscess (pharyngeal, parapharyngeal, sinusitis requiring sinus drainage, any remote abscess), acute respiratory distress syndrome (ARDS), (**c**) admission to the intensive care unit (ICU), (**d**) disease related hospital readmission and (**e**) death from any cause.

The secondary endpoints of this trial are (**a**) antibiotic exposure for LRTIs (antibiotic prescription times duration of antibiotic therapy), (**b**) side-effects from antibiotic treatment, (**c**) time to clinical stability, (**d**) length of hospital stay and (**e**) quality of life according to EuroQol and LRTI-specific disease activity score[22]. Five projects performed alongside to this trial (cost-effectiveness of PCT guided antibiotic therapy, impact of nursing and social factors for rate and duration of hospitalization, other biomarkers as diagnostic and prognostic tools in LRTI, free cortisol and copeptin levels to assess disease-related stress, microcalorimetry as a novel method for rapid diagnosis of bloodstream infections) synergize scientific efforts.

Outcomes are assessed during hospital stay at days 3, 5 and 7, and at hospital discharge and by structured phone interviews at days 30 and 180 (figure 1) by medical students blinded to the treatment allocation of the patients. In case the patient is indicating the prescription of any new antibiotic or any unnamed drug following hospital discharge or is unable to give adequate information, or has been rehospitalised, the interviewer is obliged to contact the treating GP or the hospital and to receive notification of the prescription or a copy of the hospital transferral or demission letter.

Endpoints are reassessed by an independent endpoint committee of at least 2 clinicians blinded to patient allocation. Endpoint judgment is based on the case report form and, if necessary, on hard copies of the hospital chart that are made anonymous and blinded in regard to any information on PCT. In order to truly blind endpoint assessors all information on PCT will be discarded on relevant documents.

An adverse event in a subject is defined as any occurrence of unfavourable and unintended clinically relevant medical sign, symptom, or disease temporally associated with the study which does not necessarily have a causal relationship with the study procedure. If an adverse event occurs, the responsible clinician involved in the case is contacted by an unblinded member of the study team to verify all information and to complete a serious adverse event form. All adverse events within 180 days after study inclusion are monitored and continuously evaluated by the data safety and monitoring board. All adverse events must be followed until resolution, until the condition stabilizes, until the event is otherwise explained, or the subject is lost to follow-up or has died.

### Sample size and statistical considerations

The goal of this ongoing trial is to show non-inferiority of the PCT guided antibiotic management approach in comparison to enforced guidelines for the primary endpoint (i.e. the disease-specific failure rate within 30 days). To estimate the frequency of the primary endpoint, we used the data from our previous intervention trials [11,12,14]. Based on these data, the risk of disease-specific failure in evaluable patients is assumed to be around 15% and, if losses to follow-up are treated as failures, the failure rate of all patients may increase to 20%. We assume non-inferiority if the disease specific failure for patients on procalcitonin-guided antibiotic therapy is less than 7.5% higher compared to those on guidelines-enforced management. Based on these estimates and assumptions, a minimal number of 1002 patients (501 patients per arm) are required. Table 2 displays the power calculations for different assumed failure differences and non-inferiority boundaries with a one-sided type I error of 5% and a power of 80% to 90%. With 1002 patients, we have 80% power to exclude differences in failure rates of more than 9–12% in the respective LRTI subgroups and of ≥6% in death rates. In practice, a fixed recruitment period of 18 months is considered and all patients recruited in that period be randomized unless sample size after 18 months is below 1002 patients, which would lead to an extension of the recruitment period. The target size at each centre is 250 patients completed per protocol.

**Table 2:** Required total sample size

True assumed failure rate in both arms	Required total sample size		
	Δ = 5% Power 90% (80%)	Δ = 7.5% Power 90% (80%)	Δ = 10% Power 90% (80%)

10%	1278 (932)	578 (426)	330 (244)
15%	1792 (1302)	806 (588)	458 (334)
20%	2232 (1624)	**1002 **(730)	570 (418)

Sample size calculation for the primary endpoint, i.e. required sample size to conclude at the one-sided 5%-level that the disease-specific failure rate in PCT-guided arm is at most Δ higher compared to management with enforced guidelines with a power of 80–90%.

The primary analysis population is the full analysis set which includes all randomized patients following an intention-to-treat principle. For the primary analysis, losses to follow-up (on both arms) are regarded as treatment failures. A (two-sided) 90% confidence interval for the difference of the disease-specific failure rates will be calculated based on Cochran's test stratified by type of LRTI. If the confidence interval for the difference excludes 7.5% or more, the primary objective will be met. As a sensitivity analysis, the primary analysis will be repeated on the subset of patients with evaluable outcome only, i.e. drop-outs are excluded from this additional analysis, as well as the per-protocol population which excludes non-evaluable cases and major protocol violators.

In a second step, the primary endpoint will be explored for association with potential prognostic factors in a logistic regression. The factors that will be considered are: age, sex, LRTI subgroup, PSI and CURB65 score in CAP patients and GOLD criteria and Anthonisen type of exacerbation in COPD patients [18,23]. Potential centre effects will be tested by including centre and physician as a random effect.

The trial data and sample base will be used for several pre-specified additional analyses. Theses projects include the development of clinical prediction rules for adverse medical outcomes in community-acquired LRTIs and to compare the diagnostic and prognostic accuracy of promising new biomarkers (e.g. proadrenomedullin (proADM), pro atrial natriuretic peptide (MR-proANP), copeptin, total and free cortisol) with traditional clinical signs and symptoms, the PSI and the CURB-65 score for patients with CAP and the Anthonisen criteria for patients with AECOPD [3,6,18,24-29].

## Discussion

This ongoing multicenter trial is the first prospective, randomized controlled trial in hospitalized patients with LRTI sufficiently powered to show non-inferiority of a PCT guided antibiotic management compared to enforced guidelines. This trial has the potential to demonstrate whether antibiotic stewardship based on PCT cut-off values reduces antibiotic exposure without compromising patient relevant outcomes. The present protocol should allow a more wide-spread implementation of the proposed PCT algorithm for further external validation with the aim to optimise the management of LRTIs by avoiding unnecessary antibiotic use, costs and side effects from antibiotics.

Notwithstanding the impact of PCT to improve diagnosis and antibiotic use in LRTI, its prognostic value on admission to predict complications from LRTI is limited. Other biomarkers may have better potential in the prognostic assessment of LRTIs and sepsis on admission. For example, adrenomedullin is one of the most potent vasodilating agents with immune modulating, metabolic and bactericidal properties [30,31]. Adrenomedullin precursor levels are elevated in sepsis and CAP, have a similar accuracy to predict death compared to the APACHE II score in critically ill patients and also improve the prognostic accuracy of the PSI in CAP, providing an additional margin of safety [28,29]. Another candidate biomarker is the atrial natriuretic peptide (ANP), a member of the family of natriuretic peptides that regulates a variety of physiological parameters [32]. In CAP, the level of ANP precursor peptides (MR-proANP) may mirror both, the inflammatory cytokine response correlated with the severity of pneumonia, as well as the presence of disease-relevant comorbidities, namely heart failure and renal dysfunction [33,34]. Plasma MR-proANP levels are increased in LRTI with highest levels in CAP and are better predictors of severity and outcome of CAP as compared to commonly measured clinical and laboratory parameters and comparable to the PSI [25,35]. Another promising biomarker is copeptin, stoichiometrically converted to vasopressin which has hemodynamic and osmoregulatory effects, and reflects the individual stress response [36]. Copeptin levels increase with increasing severity of CAP, as classified by the PSI score and in patients with acute exacerbations of COPD copeptin was shown to be predictive of long-term clinical failure independent of age, co-morbidity, hypoxemia and lung functional impairment in multivariate analysis [24,27,37]. The additional prognostic value of these novel biomarkers in the careful clinical assessment shall be validated within this trial for a better estimate of the likelihood for adverse medical outcomes.

Based on the large body of evidence generated by the ProHOSP study, a further trial is planned. Thereby, we plan to combine the structured clinical assessment with diagnostic and prognostic biomarkers including pivotal aspects of nursing care and social factors to conceive and implement pre-emptive early discharge measures. With the integration and validation of these new biomarkers and clinical assessment tools we expect a large potential to safely optimize health care resources. Thereby, LRTI will serve as "proof-of-concept" for other diseases.

### Potential limitations

Obviously, a state-of-the-art microbiological evaluation is necessary for decisions about appropriate step-down therapy and the use of narrow-spectrum antibiotics. However, we do not interfere with the choice of antibiotics. Routinely available methods to clarify the etiology of acute respiratory tract infections have limitations. The diagnosis of viral infection is cumbersome, expensive, delayed, and is not needed for the primary endpoint. Influenza-testing would require a cumbersome nasopharyngeal swab. Based on our data using a sensitive assay serological evidence of viral infection was found in almost 80% of assessed cases [11,12]. This rate was independent of the diagnostic subgroup of LRTI (acute bronchitis/AECOPD/CAP). However, even a positive viral serology does not rule out complicating bacterial infection. Conversely, microbial cultures are of limited value. For example, CAP is thought to be of predominantly bacterial origin. Despite thorough training, adequate sputum specimens can be obtained in only 50% of CAP. Accordingly, bacteria are identified in less than 30% and 10% of CAP-cases by sputum and blood culture, respectively [38]. Atypical pneumonias are rare, i.e., less than 5% based on PCR techniques and probably overestimated in the literature based on serologic analyses. In addition, apart from severe legionella infection, exposure to *Legionella pneumophilia *often results in seroconversion without disease. Similarly, in AECOPD positive bacterial sputum cultures are of limited use, as the majority of AECOPD patients have continuous positive sputum culture due to colonization results. Importantly, in the PCT group of a previous trial this rate was similar in patients in whom antibiotic were given or withheld, as was the outcome [14].

This protocol describes a randomized open multicenter intervention trial with an expected high external validity. However, contamination within the proposed open trial design is obvious. We expect that in a setting where physicians know that they are monitored for antibiotic use, the antibiotic prescription will be lower as compared to the real-life setting (Hawthorne effect). Similarly, adherence to the current guidelines in the guideline group may be higher as compared to the real-life setting. On the other hand, physicians may learn from their experience with PCT testing and change their clinical practice for the treatment of the guideline patients and for example may reduce antibiotic treatment or treatment duration in guideline group patients (spill-over effect). The latter bias, however, will be conservative. Thus, experience gained from treating patients according to the PCT algorithm in the intervention arm or other factors attributable to the conduct of the study (for example increased awareness by physicians because of more conscious decision making) may affect antibiotic prescription in the guideline arm and could lead to reduced prescribing or treatment duration in the control group.

It cannot formally be excluded that these biases could favour similarity of the two arms and thus be non-conservative for the primary non-inferiority comparison. However, for all secondary endpoints where superiority of the PCT group is the objective, we believe these biases to be conservative.

## Competing interests

This is an investigator driven study. No commercial sponsor had or will have any involvement in design and conduct of the studies (i.e., main project and ancillary projects of the ProHOSP study), namely collection, management, analysis, and interpretation of the data; and preparation, decision to submit, review, or approval of the manuscript.

BM has served as a consultant and received payments from Brahms to attend meetings; he received research support and fulfilled speaking engagements. All other contributors and authors have not disclosed any conflicts of interests.

## Authors' contributions

MCC and BM initiated the study, MCC, WZ, HCB, MW, PS, US, and BM designed the study and wrote the protocol. PS, US, RT, CF, IW, SN, CB, RS, CH, TB, CHH, KR, MK, WZ and BM manage the trial and collect data. PS and MW performed the statistical analyses. PS, HCB, MW, and BM drafted the manuscript. All authors amended and commented on the manuscript and approved the final version. PS, WZ, MCC, HCB, and BM oversaw the study and act as guarantor.

## Pre-publication history

The pre-publication history for this paper can be accessed here:


